# Research on the evolution of physical teachers and physical teaching policy: A perspective from China

**DOI:** 10.1371/journal.pone.0341906

**Published:** 2026-02-03

**Authors:** Mingliang Xiong, Bin Yan, Ruilin Xu, Fang Liu, Cuixiang Dong

**Affiliations:** 1 College of Physical Education and Health, East China Normal University, Shanghai, Shanghai, China; 2 School of Physical Education, Hunan First Normal University, Changsha, Hunan, China; 3 School of Mathematics and Statistics, Hunan First Normal University, Changsha, Hunan, China; 4 Jinjiang Middle School, Huaihua, Hunan, China; A'Sharqiyah University, OMAN

## Abstract

This study aims to comprehensively and profoundly explore the history of changes in China’s physical teachers and physical teaching policies. Focusing on policies related to physical teachers and teaching published between 1950 and 2023, we employed text mining and topic-matching techniques to construct an algorithmic model for analyzing physical teachers and teaching policy topic types and attitude orientations, thereby capturing the internal logic and external characteristics of the policy texts. Through a sub-period analysis, we examined the evolution of physical teachers and teaching policy themes and attitudes across different historical periods. The key findings reveal that policy themes have gradually exhibited a trend of diversification and refinement: initially emphasizing the training of physical teachers’ basic qualities, they shifted to highlighting the promotion of professional skills, and later advocated the development of comprehensive quality and innovative abilities. Concurrently, policy attitudes have transformed from being unitary to pluralistic and from rigid to flexible, with an increasing focus on guidance and encouragement. Additionally, the process of policy change demonstrates continuity and inheritance, which ensures the stability and coherence of the policy system.

## Introduction

In the tide of globalization and educational reform, physical education, as an important component of cultivating students’ physical and mental health, promoting social adaptability and disease prevention, is playing an increasingly prominent role [1]. In recent years, governments and educational institutions have issued a series of policies and measures aimed at improving the quality of physical education and promoting the all-round development of students. For example, China’s“Opinions on comprehensively strengthening and improving physical education in schools in the new era” and the United States’ “Shape America: National Standards for Physical Education” both reflect the high importance and determination to reform Physical Education. China alone has issued more than five-hundred national-level documents since 1950 that set PE teacher quotas, curriculum standards, salary supplements and professional-development requirements. These policies not only reflect the society’s re-understanding of the value of sports but also reflect the profound expectation for the professional development and teaching innovation of physical teachers [2].

However, the formulation and implementation of the Physical teacher and P.E. Policy constitute a multifaceted process, with its effectiveness being frequently influenced by a myriad of factors. These include the scientific rigor of the policy, the extent of its implementation (or policy attitude), the reception among teachers, and shifts in the social milieu [3]. Consequently, we still lack an evidence base on how policy has specifically targeted PE teachers and PE teaching. It remains unclear (i) which teacher attributes have been emphasised in each era, (ii) how policy instruments have moved from imperative mandates toward incentives or ability-building, and (iii) whether the issuing architecture is becoming sufficiently coordinated to redress rural–urban disparities in teacher supply. Consequently, a thorough analysis of the evolution of the themes and attitudes embedded within physical education policy not only aids in comprehending the internal rationale behind policy shifts but also serves as an empirical blueprint and a valuable reference for shaping future policy initiatives.

With the swift advancement of information technology and digital transformation, visualization technology has emerged as a pivotal tool across numerous domains. In this study we construct the first machine-readable corpus of 573 national-level PE teacher/PE teaching policies (1950–2023). By combining domain-validated stop-word lists, LDA topic modelling and a five-fold attitude classifier, we track thematic prominence and policy tone across three epochs. This research approach not only aids in grasping the internal logic and external characteristics of the policy texts but also furnishes a potent tool for uncovering the patterns of policy shifts.

Therefore, this paper focuses on China’s physical education teachers and physical education policy since the founding of the People’s Republic of China. Utilizing text mining, theme algorithms, and other technologies we visually present the evolution of themes and attitudes related to physical education teacher or education policy. The results offer a diagnostic mirror for agencies that fund, train and evaluate PE teachers and provide concrete entry points for targeted recruitment, redesign of professional-development pathways, and equity-oriented governance that closes teacher-quality gaps between wealthy counties and the rest.

## Literature review

Physical education teachers play a pivotal role in school physical education, and thus, research on their associated policies is crucial for enhancing the quality of education [4–6]. Since the establishment of the People’s Republic of China, state leaders have consistently emphasized the importance of physical education in schools, with Comrade Mao Zedong repeatedly underscoring its significance [7]. “The Interim Regulations on School Physical Education (https://news.hust.edu.cn/info/1002/ 45697.htm)”, jointly issued by the Ministry of Education and the Central Sports Commission, unequivocally state that the objective of physical education is to elevate students’ physical fitness and foster their holistic development. In the same year, the Ministry of Education formulated the “Physical Education Plan for Schools at All Levels and Types (https://www.360docs.net/doc/db17976057.html)”, making physical education classes mandatory. However, following 1957, the development of school physical education faced obstacles due to the Cultural Revolution. Ultimately, in 1967, military training replaced the physical education curriculum.

After the reform and opening up in 1979, the Ministry of Education, the State Sports Commission, and other departments reasserted the significance of school sports and fortified the relevant system. The Sports Law of the People’s Republic of China (https://www.sport.gov.cn/gdnps/files/c25528410/26026229.pdf), enacted in 1995, explicitly mandates that schools must establish physical education classes and integrate them into students’ academic assessment systems. In 1996, the Ministry of Education of the People’s Republic of China issued the “Overall Teaching Reform of Two Types of Physical Education Curriculum (https://www.cnki.com.cn/Article/ CJFDTotal-ZGXT199604021.htm)” to further diversify and enrich the school physical education curriculum. The triumph of the 2008 Olympic Games in Beijing ignited a nationwide passion for sports, leading to a comprehensive elevation of the status of sports teachers and physical education [8]. In 2012, the General Office of the State Council issued several recommendations aimed at further bolstering physical education in schools (https://www.gov.cn/gongbao/content/2012/content\2256572. htm), emphasizing the comprehensive enhancement of physical education in secondary schools. Recently, the Ministry of Education has consistently emphasized the need to reinforce teaching staff in weaker subjects, such as physical education and health, and has increased the compensation for special-post physical education teachers. The evolution of educational policies concerning physical education teachers in China has consistently garnered significant interest among scholars [9–11].

This paper examines the evolution of China’s physical education policy, encompassing historical shifts, societal demands, and implementation outcomes. It highlights the policy’s adaptability and challenges, while noting that limited research has focused on the policy’s attitudinal dimensions, particularly its oppressive aspects, enforcement, and moral considerations. This gap hinders our comprehensive understanding of the policy’s profound impact and obstructs policy optimization and innovation. The evolution of physical education policy mirrors not only societal shifts in the perception of physical education’s value but also the state’s dynamic adjustment of physical education teacher training goals. To broaden the research scope to the policy attitudinal level, this paper employs text analysis, theme mining models, and visualization technology to delve into the value orientations and relationships among the publishing organizations behind the policy. This approach builds a more holistic framework for policy comprehension. By exploring the historical evolution of policy themes and attitudes, we can uncover the causes, patterns, and trends of change, providing invaluable historical insights and inspiration for current and future policy-making concerning physical education teachers.

## Research design

### Methodology

#### Methods for identifying policy themes.

Policy topic identification stands as a pivotal technique in the realm of text content mining within quantitative policy research. It offers a highly effective means of summarizing the core content within a specific policy domain and demonstrates substantial application value in practical policy analysis endeavors. In this paper, we initially leverage the *jiebaR* package in R software to precisely process the policy documents. Building on this foundation, we integrate the deprecated dictionary from the Harbin Institute of Technology with manual screening to meticulously clean the data post-word segmentation, thereby guaranteeing the accuracy and validity of the dataset (Table 1). Subsequently, we employ the Latent Dirichlet Allocation (LDA) model to model the themes within the text data, successfully extracting the core themes and calculating the strength of each. Ultimately, we arrange the words in descending order based on their thematic intensity, laying a solid groundwork and providing robust support for subsequent research endeavors.

**Table 1:** Corpus cleaning pipeline.

Stage	Retained words (in Chinese)	Removed	Removed rate (%)
Initial Segmentation	5014	0	0
Stopword removal	4039	973	19.5
Manual screening (Abnormal words)	3946	93	2.3

### Methods for identifying policy attitudes

The policy attitude serves as a profound reflection of a policy’s strength and the urgency of its implementation. By conducting a meticulous analysis of word intensity in policy documents, we can select a series of frequently used attitude keywords and construct an attitude matching model to scientifically classify policy documents. It is crucial to note that any given policy document may encompass multiple distinct attitudes. Utilizing this method, we are able to visually compare and analyze the attitudes embedded within policy documents across different timeframes, thereby delving deeply into their historical characteristics and patterns of change. This approach provides us with a more comprehensive and nuanced perspective for interpreting policies.

**Fig 1:**
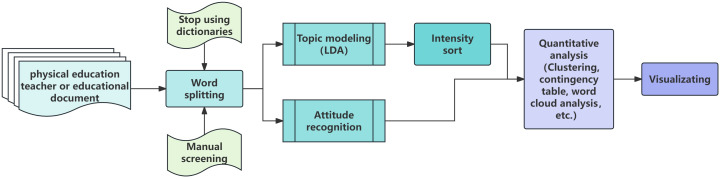
Research framework.

In conclusion, the research framework and methods of the evolution of the theme and attitude of physical education policy can be shown in Fig 1.

### Method for policy text processing

Policy documents often contain numerous references to government agencies, laws and regulations, and social work terminology. To effectively extract the core themes of these policies, it is necessary to first clean the policy text data. Initially, Sogou’s thesaurus (2012 Basic Lexicon) can be utilized to eliminate less valuable indexing information from the aforementioned three types of data within the text (Words with TF−IDF < 0.05 (normalized to max TF−IDF=1.0) were deemed “low-value” and removed.). Subsequently, the Harbin Institute of Technology’s obsolete dictionary (v3.4) can be employed to further refine and optimize the data (e.g., ‘physical exercise standard’ → ‘curriculum standard’). Ultimately, with the focus on part-of-speech data, the LDA topic model is applied to analyze and identify the main themes within the documents.

To ensure topic robustness across repeated runs, we implemented:

- LDA was executed 10 times with random initializations for each topic number;
- We calculated the Jaccard similarity index for topic keywords across runs. Topics with ≥ 80% keyword overlap were deemed stable;
- Quantified stability via ‘Topic Stability Score (TSS)’:

$$\text{TSS}=\frac{\text{1}}{R}\sum {\mathrm{\backslash nolimits}}_{r=\text{1}}^{R}\frac{{\text{Consistent}\;\text{Topics}}_{\_\text{r}}}{\text{Total}\;\text{Topics}}.$$

After running the LDA model 10 times, the average TSS: 0.89 (threshold 0.85) was obtained, proving that the LDA model is stable in this scenario.

### Data sources

The study was centered on policies related to physical education and teaching, issued between 1950 and 2023 (The deadline for document retrieval is December 31, 2023.) at both the Central and state ministry levels. It is important to acknowledge that fewer policy documents specifically address the education or teaching of physical education teachers compared to those for all teachers. However, since physical education teachers are included within the broader category of all teachers, some policy documents, while not explicitly mentioning physical education teachers, still have a direct impact on their pre-service and post-service development, as well as related rights and interests. Consequently, these policies are also encompassed within the scope of this study.

Guided by the three principles of openness, authority, and systematicness in gathering policy documents, and with the aim of ensuring the scientific and reliable nature of the research findings, this study drew heavily on previous research outcomes. Employing the “Semantic search” method with keywords such as “Physical education”, “Physical teacher”, “Teacher”, “Education”, and “Teaching”, we retrieved 769 policy documents from the CNKI (https://lawpro.cnki.net/urtp-cli/advsearch? sysid = 7000\&res = clkl). To complement these, we further searched the Chinese Government Net State Council bulletin (https://www.gov.cn/zhengce/zhengce wenjianku/) using the same keywords and identified an additional 113 policy documents pertinent to physical teacher and education.

### Data collection and screening

Expert Research Protocol:

- **procedure:** Three domain experts (two professors of physical education policy with 15 + years of research experience, and one senior policy analyst from China’s Ministry of Education) independently reviewed the titles and abstracts of all 882 initial documents.
- **Criteria:** Documents were flagged for exclusion if they: (a) Were administrative notices (e.g., meeting schedules, funding announcements) without substantive policy content; (b) Duplicated identical policies across different government portals; (c) Predated 1950 (outside our temporal scope of 1950–2023).
- **Inter-rater Reliability:** Cohen’s κ=0.82 after initial review, with discrepancies resolved through consensus meetings.

Content Study Methodology:

- Explicit mention of tertiary physical education systems;
- Specification of teacher qualifications/training requirements;
- Inclusion of curriculum standards or assessment frameworks;
- Reference to resource allocation (facilities, funding, staffing).

Four research assistants identified the effectiveness of the collected policies based on the four criteria mentioned above and retained their common elements.

Subject Matching Mechanism:

- **Algorithmic Pre-screening:** We employed TF-IDF vectorization to compute keyword density for core concepts (“Physical education”, “Physical teacher”, “Teacher”, “Education”, and “Teaching”). Documents with aggregate scores below 0.35 (normalized to max = 1.0) were automatically excluded.
- **Human Validation:** The algorithm’s output was verified against manual assessments by our expert team (accuracy: 94.7%; false positives reviewed individually).

Following this, through the application of “Expert research,” “Content study,” and “Subject matching” methodologies, we screened out unqualified documents, ultimately culminating in a final dataset of 573 policy documents. Based on this refined dataset, the paper establishes a corpus of pyhsical teacher and education texts and proceeds with subsequent research on policy evolution.

## Data Processing

### Policy theme model construction

Policy thematic (or topic) modeling is an intricate and elaborate process that encompasses the identification, categorization, and delineation of attributes and behaviors pertaining to various roles within the policy system. Prior to constructing the policy theme model, it is imperative to elucidate the comprehensive requirements of the policy system. This necessitates an understanding of the policy’s objectives, the domains it encompasses, and the stakeholders involved. Having clear requirements simplifies the subsequent categorization of topics and the determination of attributes. Consequently, in order to establish the theme evolution model for the physical teacher and education policies, we must first classify the policy themes. This classification can be approached from two perspectives: the type of policy title and the type of policy tool, as detailed in Subsection “Data source”.

### Policy attitude evolution Model

Attitude serves as an evaluative inclination that can exert both indicative and dynamic influences on all facets of a subject’s responses. The government, through the promulgation of policies, authoritatively allocates benefit relationships among various subjects. Embedded within policy texts, policy attitude shapes, alters, and sustains relationships among subjects through the linguistic constructs employed. The majority of existing research on textual attitudes is grounded in appraisal theory, which involves encoding, identifying, and statistically analyzing attitudes within texts. From a discourse analysis standpoint, appraisal theory emphasizes attitudes, emotional intensity, values expressed in the text, and various strategies for engaging readers. It plays a pivotal role in constructing the ideological foundation of discourse, evaluating, and organizing discourse structure in text analysis. Consequently, this paper utilizes a word-matching algorithm to classify policy tools spanning from 1950 to 2023 and calculates the proportion of attitudes among these policy tools for the year 2023.

## Results

### Visual analysis of policy for physical teachers or physical education across different time periods (or stages)

#### Phase division of policy themes.

After the founding ceremony of the People’s Republic of China, social order gradually stabilized, and physical education flourished amidst the robust development of education. The promulgation and implementation of a series of policies pertaining to physical education teachers and teaching have significantly contributed to our nation’s physical education landscape. The formulation and enforcement of these policies reflect the unique historical context and demands of their respective eras or environments. Therefore, research into the characteristics and evolving patterns of the subject matter within physical education policies across different time periods is of considerable interest. Consequently, delineating the evolutionary trajectory of physical teacher and education policy, as illustrated in Table 2, is of great significance.

**Table 2:** Division of physical education policies.

Literatures	Data span	Phase I	Phase II	Phase III	Phase IV
[12]	1949-2020	Genesis stage 1949-1977	Adjustment stage 1978-1997	Transition stage 1998-2008	Intensive stage 2009-2020
[13]	1949-2020	Infancy stage 1949-1977	Reconstruction and expansion stage 1978-1998	Harmonized development stage 1999-2020	
[13]	1949-2020	Political orientation stage 1949-1977	Economic orientation stage 1978-1999	Teacher Orientation stage 2000-2020	
[14]	1949-2014	Pre-reform and development stage 1949-1977	After reform and development stage 1978-2014		
[15]	1949-2014	Planning stage 1949-1977	Rule of law stage 1979-2014		
[16]	1949-2019	Early stage of development 1949-1977	Bring order out of chaos 1978-1989	Gradually improving 1990-2000	Rapid development 2001-2019

Undoubtedly, studying the evolutionary path of policy topics and exploring and analyzing their core connotations constitutes a highly insightful method. Policy topics, being the concise summaries and quintessences of policy texts, frequently mirror the core concerns, objectives, and implementation priorities of policies. Therefore, by extracting the subject matter of physical teacher and educational policy and dividing it into phases based on temporal dimensions, we can more precisely grasp the fluctuations of the policy subject, unveiling its inherent logical relationships and evolutionary trends.

This section focuses on the subjects of physical education policies issued by the Chinese state-level authorities during the period from October 1949 (considered as part of 1950 for convenience) until the present day, encompassing the major stages in the evolution of physical teacher and education policy since the founding ceremony of the People’s Republic of China in 1949. Over this extensive historical period, as the country’s political, economic, cultural, and educational landscapes have continually evolved, the education policy for physical education teachers has undergone various stages, including initial exploration, gradual standardization, and comprehensive deepening. By mining the themes of these policy topics, we can clearly discern shifts in policy focus, adjustments to policy objectives, and innovations in policy methodologies.

**Fig 2:**
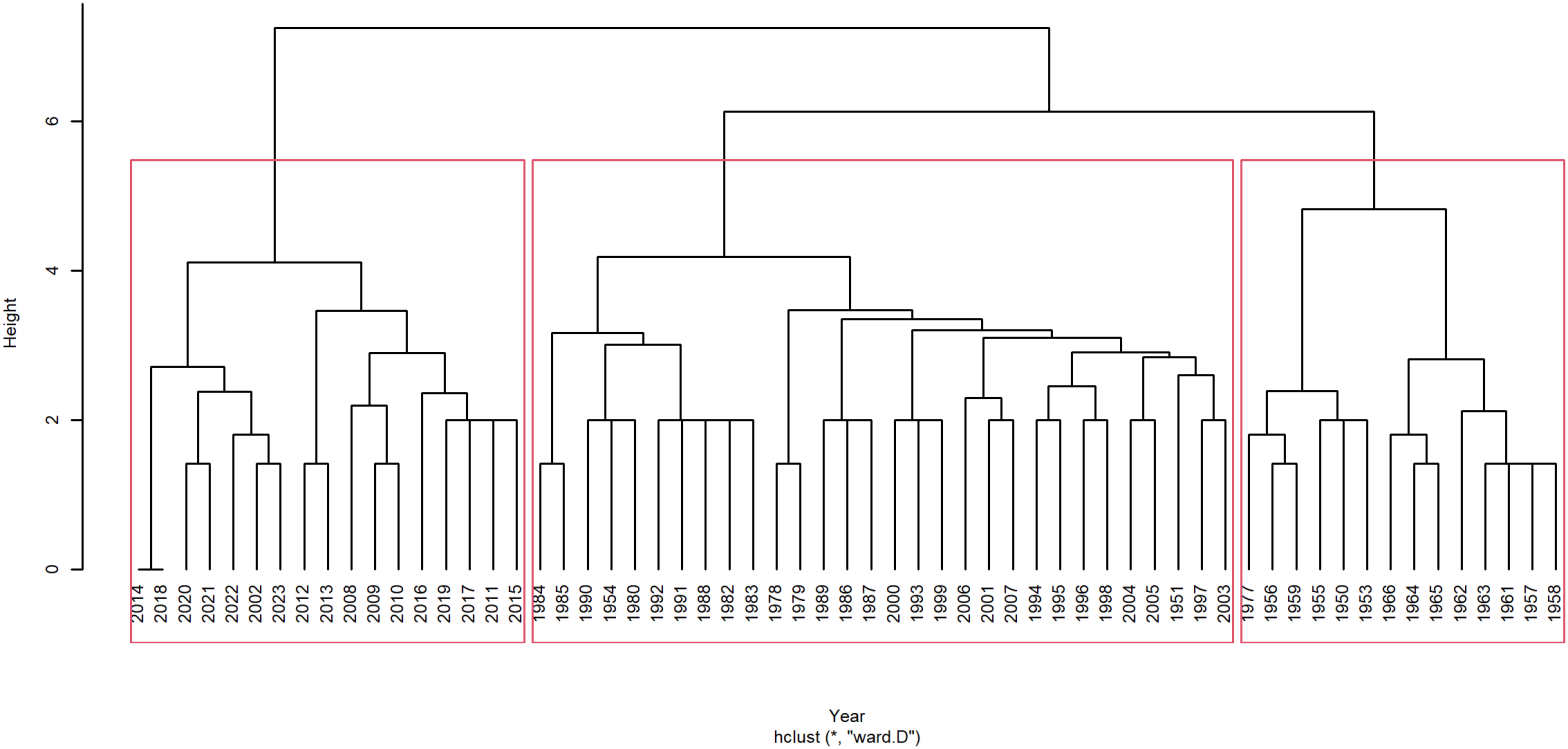
Clustering of physical educational policies in time dimension.

In the specific research process, our initial step involves collecting and organizing all pertinent physical teacher and education policy documents from the designated period. Subsequently, we employ natural language processing and text mining technologies to extract policy topics through keyword identification. To underscore the significance of the policy topic themes, we retain only the three most frequently occurring keywords for each year. Following this, we calculate the relevance of these keywords and ultimately cluster them based on temporal indicators, as illustrated in Fig 2.

To mitigate the arbitrariness in selecting the cluster of *K* (Number of clusters), we employed the Dunn index, a cluster validity index (CVI) for clustering evaluation. Introduced by J.C. Dunn in 1974, this metric aims to identify cluster configurations that are both compact(exhibiting small variance within clusters) and well-separated(where the distances between different cluster means are substantial compared to the intra-cluster variance). A higher Dunn index value signifies better clustering performance. The Dunn Index (DI) is defined as:


$$Dunn(K)=\frac{\underset{\text{2}\leq i<j\leq K}{\text{min}}d\left(C_{i},C_{j}\right)}{\underset{\text{2}\leq l\leq K}{\text{max}}△\left(C_{l}\right)},$$

where $d\left(C_{i},C_{j}\right)$ represents the distance (or separation) between clusters $C_{i}$ and $C_{j}$, $△\left(C_{l}\right)$ is the diameter (or maximum intra-cluster distance) of cluster $C_{l}$.

**Fig 3:**
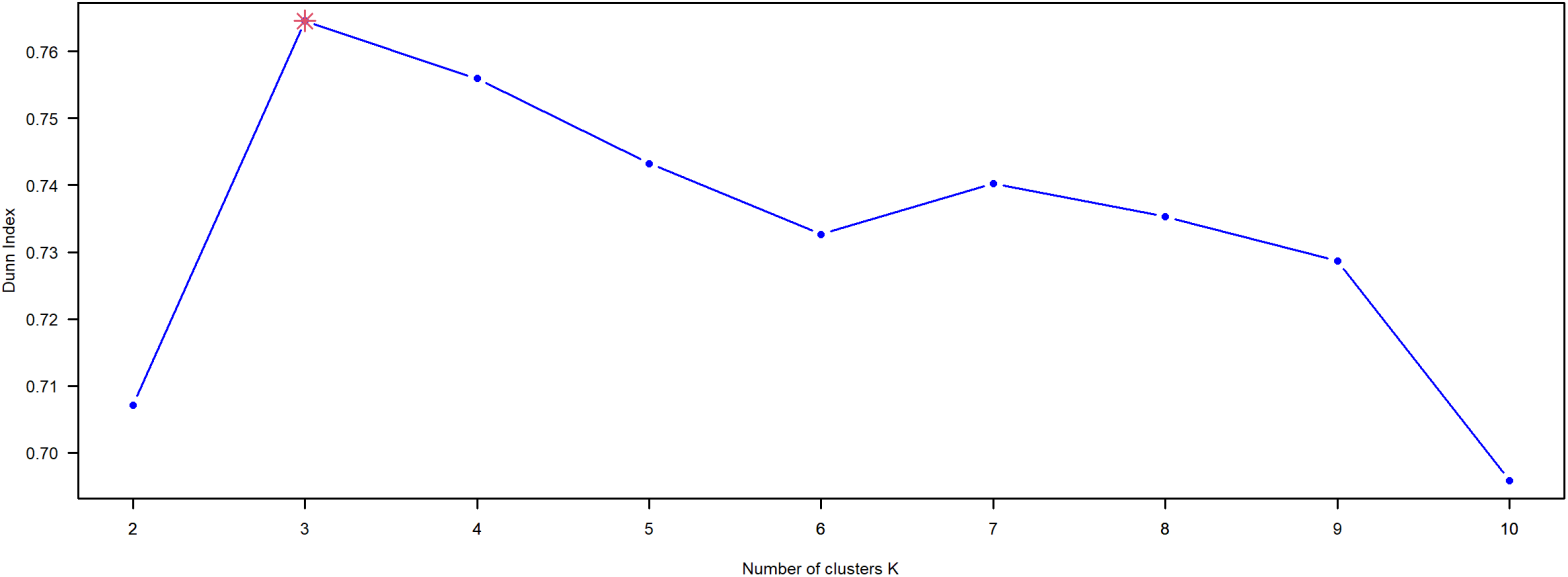
Dunn index score.

**Fig 4:**
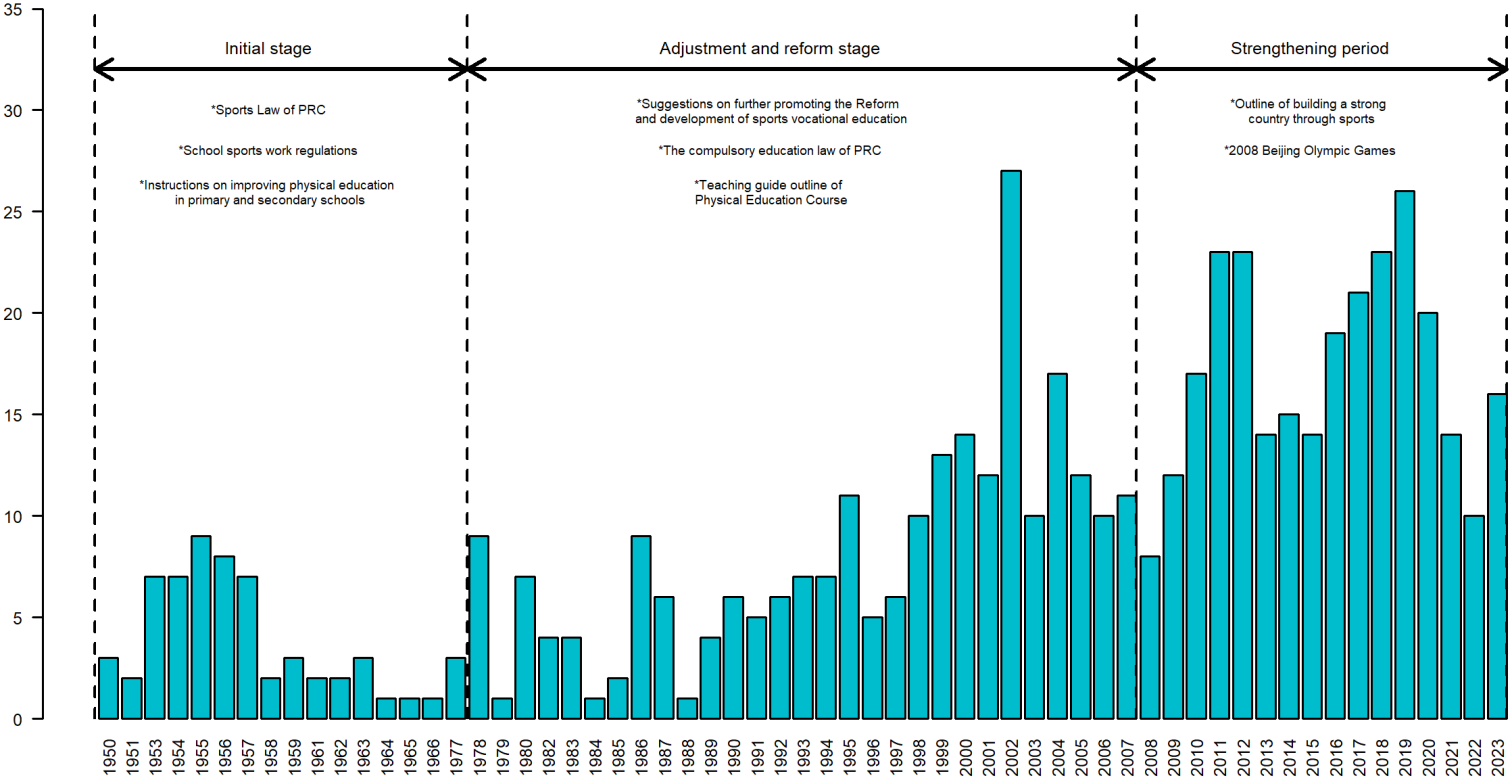
Policy annual output and stage division (1950-2023).

We computed the Dunn score for each candidate *K* using ‘clValid’ package in R. The topic stability for K=2,3,⋯,10, and for the optimal K=3, the Dunn coherence score is 0.71 (see Fig 3), which is the max value among the results. Thus, the physical teacher and education policy from 1950 to 2023, can be broadly categorized into three main types, as presented in Table 3. Building upon this foundation, we integrate historical context and social transformations, along with an in-depth examination of the characteristics of the policy themes at each stage, the reasons for change, and their societal impacts. Consequently, we divide the policy topics into three sequential phases in chronological order, as illustrated in Fig 4.

**Table 3:** Division of physical education policies.

Class	Year
1	2002, 2008, 2009, 2010, 2011, 2012, 2013, 2014, 2015, 2016, 2017, 2018, 2019, 2020, 2021, 2022, 2023
2	1951, 1954, 1978, 1979, 1980, 1981, 1982, 1983, 1984, 1985, 1986, 1987, 1988, 1989, 1990, 1991, 1992, 1993, 1994, 1995, 1996, 1997, 1998, 1999, 2000, 2001, 2003, 2004, 2005, 2006, 2007
3	1950, 1953, 1955, 1956, 1957, 1958, 1959, 1961, 1962, 1963, 1964, 1965, 1966, 1977

**Initial stage (1950–1977)** the policy framework for physical education started to take shape progressively. Notable milestones during this period encompassed the enactment of sports legislation and the promulgation of school sports regulations.

**Adjustment and reform stage (1978–2007)** At the dawn of China’s reform and opening-up era, physical education began to align with Western standards. Key milestones during this period included the “Further Promotion of the Reform and Development of Sports Vocational Education” and the issuance of the “Guidelines for the Teaching of Sports Courses.” These initiatives significantly enhanced the professional and teaching competencies of physical education teachers, thereby fostering the holistic development of physical education in China.

**Strengthening period(2008–2023)** Beijing’s successful hosting of the 2008 Olympic Games sparked a national sports upsurge. During this time, the sports education policy gained a greater emphasis on details and operability, addressing aspects such as the evaluation of sports teachers’ professional titles and their treatment. With the gradual advancement of information and modernization, the release of “The Outline of the Construction of a Strong Sports Country” and other relevant policies marked the official progression of China’s physical education into a phase of enhanced construction.

The steady rise in the number of publications reflects the gradual refinement and deepening of China’s education policy for physical education teachers. Policy-makers consistently draw lessons from experience, adjusting and optimizing policy content to ensure its relevance and effectiveness in response to the evolving needs of the times and social changes. Fig 4 illustrates a consistent increase in the number of policy papers across the three stages, aligning with the development trajectory of physical education in each phase. In the nascent days following the founding ceremony of the People’s Republic of China, the nation’s education system was in a phase of comprehensive recovery and reconstruction. However, constrained by the economic development level and societal conditions of the era, policy formulation and implementation efforts were relatively limited. As the reform and opening-up process deepened, the number of publications significantly increased, with policy-making becoming more frequent and meticulous. The economic system reform and the burgeoning market economy created favorable conditions for the reform and development of physical education. Additionally, the trend of education internationalization and advancements in science and technology necessitated adjustments and improvements to the physical education teacher policy to cater to the demands of the new era. In the third stage, with a focus on balanced educational development and the enhancement of teaching staff construction, the education policy for physical education teachers emerged as a pivotal issue in the educational sphere. The government consistently augmented the number of publications during this period by introducing and refining pertinent policies to propel the modernization and internationalization of physical education, while bolstering their professional literacy and teaching prowess. Policy formulation became more comprehensive and in-depth.

### The evolution of the system coordination network of policy-issuing organs

**Fig 5:**
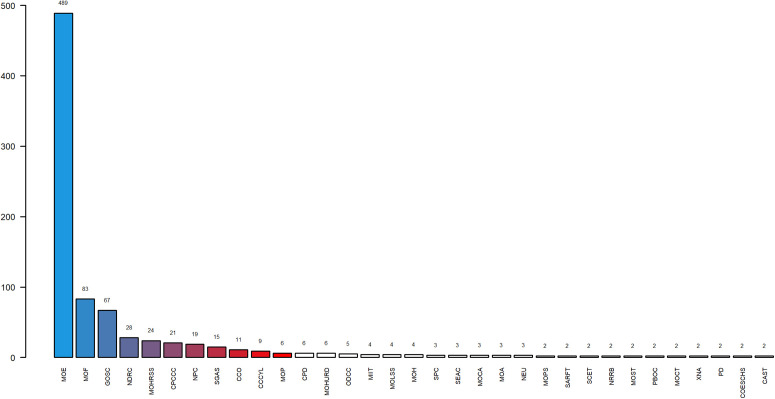
Number of policies issued by agencies.

The subjects of 573 policy documents were extracted and categorized, encompassing a total of 57 government agencies. For the visual analysis of the follow-up results, we have abbreviated the names of the institutions and Table 4 shows the abbreviations and full names of the 57 institutions. Please note that some institutional names have undergone changes over time. For instance, the General Office of the State Council (GOSC) was previously known as the State Council of the Central People’s Government prior to 1954, the Ministry of Education (MOE) before 1988 was called the National Education Commission, and the State General Administration of Sports (SGAS) was called the National Sports Commission before 1998. The policy documents issued by these renamed organizations prior to their renaming, we will allow them to be marked by their current names. To provide a clearer visualization of the distribution of authoring institutions, Fig 5 specifically highlights those agencies that have released more than two documents. As evident from Fig 5, among these 57 agencies, 33 have issued more than two publications. Notably, the Ministry of Education (MOE) stands out with the highest number of physical teacher and education policies, totaling 489, which underscores its pivotal role in the formulation and dissemination of physical teacher and education policies. Following closely is the Ministry of Finance (MOF) with 83 policies, indicating its significant influence in educational funding investments, financing policy planning, and ensuring teachers’ welfare. The top five also include the GOSC, the National Development and Reform Commission (NDRC), and the Ministry of Human Resources and Social Security (MOHRSS). The active involvement of these institutions further exemplifies the diversified and comprehensive nature of physical teacher and education policy-making, spanning various crucial aspects such as policy planning, financial backing, human resource allocation, and more.

**Table 4:** Abbreviations and full names of organizations.

Abbreviation	Full name	Abbreviation	Full name
ACFTU	All-China Federation of Trade Unions	MOPS	Ministry of Public Security
CAOS	Chinese Academy of Sciences	MOSS	Ministry of Social Security
CAST	China Association for Science and Technology	MOST	Ministry of Science and Technology
CBRC	China banking regulatory commission	MOVA	Ministry of Veterans Affairs
CCCYL	Central committee of the communist youth league	MOWR	Ministry of Water Resources
CCO	Central Compilation Office of PRC	NDRC	National Development and Reform Commission
CDPF	China Disabled Persons’ Federation	NEU	National Education Union
CFA	Chinese Football Association	NFA	State Forestry Administration
COESCHS	Committee of Education, Science, Culture, Health and Sports	NGA	National Grain Administration
CPCCC	The Communist Party of China Central Committee	NHC	National Health Commission
CPD	Central Propaganda Department of PRC	NHSA	National Healthcare Security Administration
CRTU	Central Radio and Television University	NLC	National Language Commission
CSRC	China Securities Regulatory Commission	NPC	National People’s Congress
CWF	China Womens Federation	NRRB	National Rural Revitalization Bureau
GOSC	General Office of the State Council	NSC	National Science Council
GPDOCPLA	General Political Department of the Chinese People’s Liberation Army	NYCFWLG	National Youth Campus Football Work Leading Group
MIIT	Ministry of Industry and Information Technology	ODCC	Organization Department of the CPC Central Committee
MOA	Ministry of Agriculture	PBOC	People’s Bank of China
MOCA	Ministry of Civil Affairs	PD	Publicity Department
MOCT	Ministry of Culture and Tourism	PLAGSD	People’s Liberation Army General Staff Department
MOE	Ministry of Education	SAIC	State Administration for Industry and Commerce
MOF	Ministry of Finance	SAOT	State Administration of Taxation
MOH	Ministry of Health	SARFT	State Administration of Radio, Film, and Television
MOHRSS	Ministry of Human Resources and Social Security	SCET	State Commission for Economics and Trade
MOHURD	Ministry of Housing and Urban-Rural Development	SEAC	State Ethnic Affairs Commission
MOJ	Ministry of Justice	SGAS	State General Administration of Sports
MOLR	Ministry of Land and Resources	SPC	State Planning Commission
MOLSS	Ministry of labour and social security	XNA	Xinhua News Agency
MOP	Ministry of Personnel		

**Fig 6:**
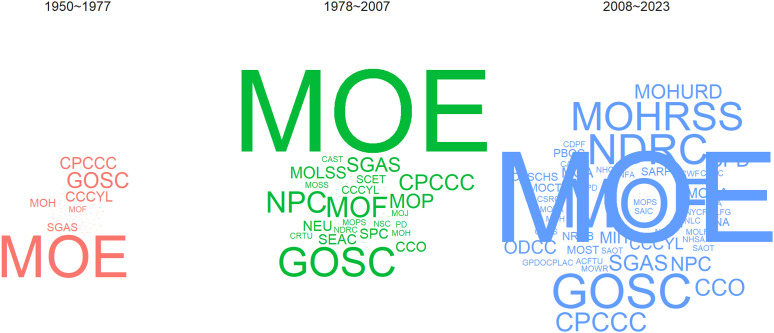
Cloud map of policy-issuing agencies.

To comprehensively unveil the dynamic evolution of the publishing institutions responsible for physical teacher and educational policies, we have meticulously conducted a phased word cloud analysis of the publishing entities (refer to Fig 6). Upon scrutinizing Fig 6, it becomes apparent that across three distinct stages, the MOE has consistently occupied a central and leading role in publishing physical teacher and education policies, while the GOSC has maintained a pivotal position. Overall, the trend in the evolution of authoring institutions is marked by an expanding scale, particularly in the third stage, where entities such as the NDRC, the MOHRSS, and other departments have actively engaged in formulating policies for physical teacher and education. This notable shift not only fortifies the robust development of sports but also fully aligns with the aspirations of sports teachers for rights protection and needs fulfillment.

From the perspective of agency autonomy, the primary entities involved in policy publication concerning physical teacher and education can be distinctly categorized into two groups: independent publication and joint publication. Table 5 outlines the distribution of agencies that have issued a total of 573 policies on physical teacher and education since 1950. Notably, 439 of these policies are independently issued, while 134 are jointly issued. The number of independently issued documents is nearly 3.3 times that of jointly issued documents. This data distribution, to some extent, indicates a lack of collaboration among various institutions in the realm of physical teacher and education policy-making in our country, often referred to as the “multi-door” phenomenon.

**Table 5:** Policy-issuing agency.

Stage	Independent(percent%)	Joint(percent%)	Mean_Joint	Total
1950-1977	55(90.16%)	6(9.84%)	0.33	61
1978-2007	198(83.54%)	39(16.46%)	1.30	237
2008-2023	186(67.64%)	89(32.36%)	5.56	275
Total	439	134	–	573

**Fig 7:**
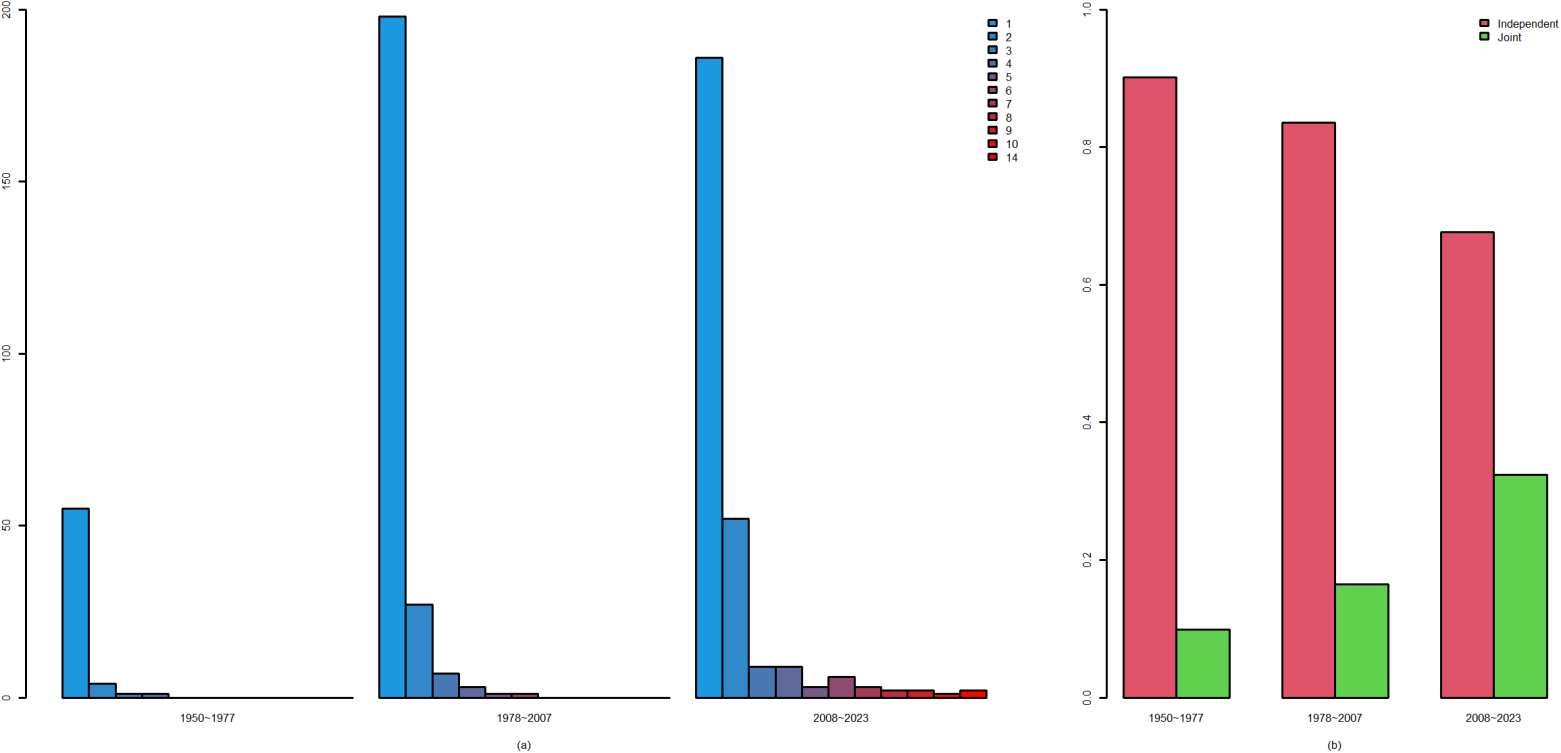
Policy-issuing agency in stages. (a): The number of agencies that co-authored the physical teacher and education policy. **(b)**: The proportion of independent and joint agencies at each stage.

A deeper analysis of independent and joint publications at each stage, as depicted in Fig 7(b), reveals a clear trend: The proportion of independent publications is steadily declining, whereas the momentum of joint publications is growing stronger. Specifically, according to Table 5, co-published policies accounted for only 9.84% of the total in the first stage, rising to 16.46% in the second stage, and reaching 32.36% in the third stage. Concurrently, the annual average number of joint publications increased from 0.33 in the first stage to 5.56 in the third stage.

This series of changes underscores a growing appreciation among policy-making institutions for the significance of collaboration and a corresponding increase in collaborative policy-making practices over time. Such a positive shift will not only facilitate the integration of resources from diverse sectors and fields but also enhance the scientific, coherent, and effective nature of policies. Consequently, this trend provides more robust policy support for the continued healthy development of physical teachers. Therefore, this trend signifies not only the optimization of policy-making but also marks an important milestone in the evolution of our educational policy system towards greater coordination and efficiency.

When examining the number of agencies that co-authored the physical teacher and education policy, as illustrated in Fig 7(a), it becomes evident that the trends significantly mirror the intensifying process of collaboration among government agencies. In the initial phase, the number of agencies issuing joint communications peaked at four, which, while indicating a nascent sense of multisectoral linkage, was still relatively limited in scope. During the second phase, this phenomenon further evolved, with a maximum of six agencies participating in the publication, signaling an expansion in both the breadth and depth of cross-sectoral collaboration. By the third stage, the number of agencies issuing joint communications achieved a qualitative leap, reaching a peak of 14 agencies—a remarkable surge that not only underscores the burgeoning synergy among government agencies but also signifies that information sharing, resource integration, and complementary advantages have become standard practices in policy formulation and project implementation.

This extensive and in-depth cooperation undoubtedly enhances the efficiency of government work, optimizes resource allocation, and provides robust support for swiftly responding to societal needs. Moreover, this trend also indicates a positive transformation in the government’s governance model, transitioning from traditional fragmentation and separation towards holistic and collaborative governance. With the increased participation of more institutions and deeper cooperation, government services will benefit the populace more efficiently and precisely, fostering comprehensive and sustainable socio-economic development. Thus, the augmentation in the number of joint agencies is not merely a quantitative change but also a vivid reflection of the profound shifts in government governance philosophy and practice.

### Visual analysis of the evolution of physical teacher and education policy theme mining

The policy theme stands as the cornerstone of the policy implementation process, directly mirroring the policy areas of focus and their hierarchical priorities. Generally, the more frequently a particular policy theme emerges within a specific timeframe, the greater the implementation efforts and attention devoted to it. This provides us with a crucial benchmark for assessing the effectiveness and impact of policy implementation. There are two prevalent approaches to exploring and comprehending policy thematic patterns: Firstly, analyzing the types of policies within policy headings, which constitute the fundamental elements of policy documents, and often succinctly encapsulates the core content and objectives of the policy. By meticulously examining policy headings, we can swiftly discern the policy type, which is frequently intertwined with policy attitudes. This foundation allows us to further dissect policy attitudes. Secondly, analyzing the types of policy instruments—the specific means and methods employed by the government to attain defined policy objectives—forms the bedrock of policy implementation. Each type of policy instrument possesses distinct advantages and constraints when addressing different issues. Hence, by scrutinizing the types of policy tools, we can gain a deeper insight into the specific implementation of policies and strategies, along with the mechanism of action across various domains and levels of policy.

Based on their nature and requirements, the topics (themes) found in the titles of current physical teacher and education policy documents in China are categorized into five distinct levels, as detailed in Table 6. In terms of attitude expression, these topics exhibit a trend of gradual change from strong to soft.

**Table 6:** Title type of policy for physical teacher and education.

Directive Policy	Normative Policy	Informative Policy	Planning Policy	Recorded Policy
Decision, Instruction, Opinion, Guide, Instruction, Outline, Strategy	Stipulate, Rule, Method, Regulations, Standard, Bylaw, Law	Notice, Letter	Project, Plan, Scheme, Design, Draft	Notes, Reports

At the top of the hierarchy is the directive policies, representing the highest level in the system. It is resolute and strict, unequivocally mandating strict adherence to the document’s requirements. Its concrete forms include the decision, instruction, opinion, guide, instruction, outline, or strategy, all possessing a high degree of authority and enforceability.

Following the directive is the normative policy, meticulously crafted by authorities to establish universally binding norms and standards. These provide clear guidance and rationale for policy implementation.

Informative policies play a pivotal role in conveying information and overseeing implementation. They alert lower-level departments to the contents of directives or normative documents, ensuring the effective dissemination and enforcement of policy spirits.

Planning policies, characterized by flexibility and adaptability, focus on the current or future design and discussion of specific themes. They can adjust and optimize based on feedback during actual implementation, ensuring the smooth attainment of policy objectives.

Lastly, serving as the foundation of the system, recorded policies primarily record important leaders’ speeches, talks, and the outcomes of crucial meetings and discussions. These records are not merely historical reenactments but also crucial carriers of policy continuity and innovation.

On an international scale, the categorization of policy instruments exhibits a diversity of characteristics. To more accurately explore the feasibility of teaching policies for physical teachers, we have adopted the classification method proposed by Schneider and Ingram [17], taking full consideration of the authority and applicability of policy tool classifications. Schneider and Ingram’s categorical framework not only offers insights into the perception of government as a power subject but also considers the reality of the target group, the inherent attributes of policy tools, and their optimal application conditions in different contexts. This classification demonstrates strong explanatory power in the realm of education policy, focusing on analyzing the relevance and adaptability of various policy instruments to specific educational issues. Consequently, it has been widely recognized and favored by education policy researchers. Under Schneider and Ingram’s classification framework, physical teacher and education policies are meticulously divided into five categories (see Table 7). This classification not only deepens our understanding of the nature and characteristics of teaching policies for physical teachers but also provides policymakers with more precise and practical tool selection guidelines, thereby promoting the continuous improvement of physical education quality.

**Table 7:** Types of policy tools for physical teacher and education.

Type	Authority	Incentive	Persuasion	Ability-building	System reform
Concept	Binding or regulating the behavior of individuals and institutions through absolute obedience to rules	Inducing the object to comply with an action through a tangible reward by communicating a vision or value.	Restrict or promote the behavior of individuals, institutions	Helping individualsor organizations improve their capacity by providing training, educational resources, etc.	Adoption of asystematic and comprehensive reform program to regulate the behavior of institutions or individuals
Typical words	Must, Forbid, Require, Implement, Obey, etc	Investment, remuneration, resources, wages, etc.	Encourage, Propagandize, Study, Attract, Guide, Promote, Strengthen, etc.	Training, Further education, education, etc.	Reform, improvement, rectification, elimination, improvement, etc.

### Policy theme strength analysis

**Fig 8:**
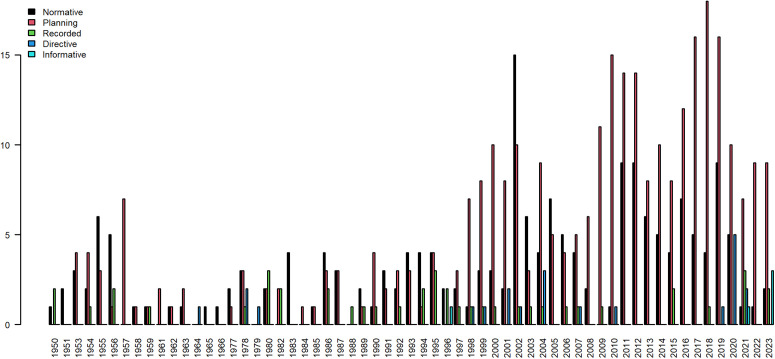
Number of policy types over the years.

In an in-depth exploration of the evolution of physical education policies from 1950 to 2023, we conducted a meticulous classification of the 573 policy titles issued over this 73-year span. These classifications were precisely matched based on the carefully selected keywords outlined in Table 6. The results of this classification exercise, depicted in Fig 8, visually illustrate the trends in the number of various policy types. It is evident from the graph that the quantity of documents, regardless of type, exhibits a consistent upward trajectory. This pattern unequivocally demonstrates the sustained and deepening commitment of governmental bodies to managing the field of physical education, underscoring the paramount importance attached to enhancing the quality of physical education and fostering the professional growth of teachers.

**Fig 9:**
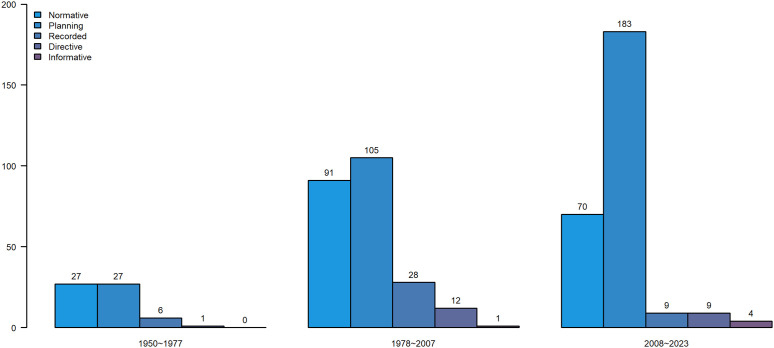
Number of policy types at different stages.

Fig 9 presents a detailed breakdown of the absolute number of changes in physical teacher and education policies issued between 1950 and 2023, categorized into three distinct stages. This chart not only furnishes us with intuitive data regarding the frequency of policy releases but also unveils the trends and evolution of diverse policies across different historical epochs. A careful inspection of the graph reveals a consistent increase in the number of recorded and informative-type policies across all three stages. This upward trajectory suggests an increasing emphasis on documenting, summarizing, and reflecting on the educational activities of physical teachers, in tandem with the progression of educational practices. Moreover, the informative policies may signify a continuous augmentation in the information dissemination and sharing capabilities of governmental bodies. Through these visual policies, the latest educational philosophies, teaching methodologies, and policy-oriented insights are promptly communicated to the vast majority of physical teachers, thereby fostering continuous innovation and optimization in educational practices.

In contrast, when compared to the first stage, the second stage witnesses an upward trend in the absolute numbers of normative, planning, and Directive-based policies. This shift may indicate that, during a specific historical period, the government formulated a series of related policies aimed at strengthening the standardized, planned, and directive management of physical education. However, by the third stage, the number of all three types of policies had declined to varying degrees. Notably, the decline in directive-type policies is particularly pronounced. This decrease may reflect a shift in the governmental approach to education policy-making, transitioning from a past emphasis on rigid management characterized by order and obedience to a more flexible guidance focused on adaptability, innovation, and responsiveness.

The decline in normative and planning documents could suggest that rigid norms and plans are progressively being supplanted by more adaptable management strategies within educational practice. As educational reform intensifies, the formulation and execution of educational policies for physical teachers increasingly prioritize alignment with real-world conditions. Consequently, educators are encouraged to adapt and innovate in response to shifts in both internal realities and external environments, ultimately aiming to achieve continuous enhancement in the quality of education.

### Theme evolution path and trend analysis

**Fig 10:**
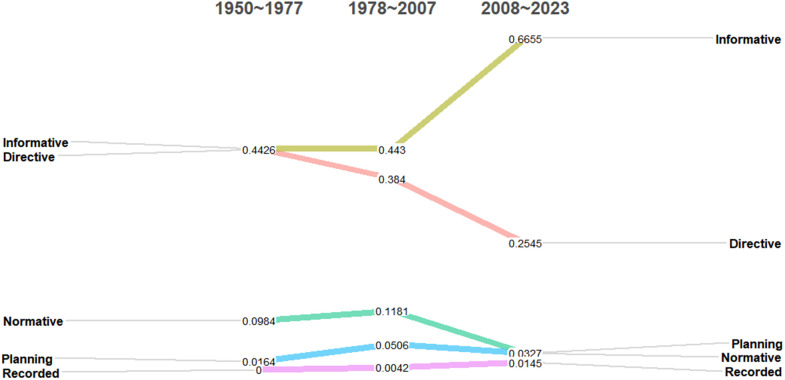
Evolution of the proportion of policy types at different stages.

Furthermore, Fig 10 depicts the evolution of the proportion of educational policies for five categories of physical teacher and education across different historical stages (categorized into three distinct periods). This chart clearly illustrates that directive and informative policies have consistently occupied a dominant position among the five types. However, the developmental trends of these two policy types diverge significantly: the proportion of informative policies has consistently risen over time, particularly in the third stage, where it peaks at 66.55%, surpassing the combined total of the other four document types. This significant figure underscores the increasing emphasis by government departments on information dissemination, sharing, and policy-oriented guidance within educational policymaking. Conversely, the proportion of directive-type policies exhibits a notable decline, from 44.26% in the initial stage to 25.45% in the current stage. This transformation reflects a shift towards a more lenient and open approach by government departments in formulating policies for physical education, transitioning away from the past emphasis on strict adherence to mandatory directives and moving towards a more flexible and constructive policy orientation. Encouraging educators to adapt and innovate according to their unique circumstances is crucial in this new landscape. Among the remaining three types of physical teacher and education policies, the proportion of record-based policies shows a gradual and steady increase, indicating an increasing focus on documenting and summarizing educational processes and outcomes. Meanwhile, normative and planning policies, despite a brief surge in the second stage, plummet to a low of approximately 3% in the third stage. This may suggest that in policy practice, rigid norms and plans are progressively being replaced by more adaptable management strategies.

In summary, these data not only underscore the transformation in the attitude of government departments towards policymaking for Physical education, shifting from rigid management to flexible guidance, but also hint at a future focus in physical teacher and education policy on flexibility, adaptability, and innovation. This shift aims to better support the professional development of physical teachers and ensure the sustainable growth of physical education.

## Visual analysis of physical teacher and education policy attitude

### Polar comparison of policy attitudes

Policy attitude holds a pivotal position in the policymaking process. It dictates the direction and emphasis of policy, shaping its content, form, and the ultimate impact of its implementation. When policy attitude is clear and positive, it motivates policy executors to diligently promote policy implementation and ensure the achievement of policy objectives. Conversely, a vague or negative policy attitude may demotivate policy implementers, leading to weak policy enforcement.

By aligning the segmentation results from the policy text with the classical terms listed in Table 7, we can derive the classification results for China’s educational policy attitudes towards physical teachers since the founding of the People’s Republic of China, as presented in Table 8. It is important to note that a single policy document may contain multiple policy attitudes. Whenever a term from Table 7 matches an attitude in the policy document, we increment the count for the corresponding attitude type by one. However, if multiple attitude words of the same type appear within the same policy, they will only be recorded once for that specific attitude type.

**Table 8:** Distribution of policies attitude.

Stage	Authority	Incentive	Persuasion	Ability-building	System reform
1950-1977	61(23.64%)	32(12.42%)	61(23.64%)	60(23.25%)	44(17.05%)
1978-2007	227(21.47%)	182(17.22%)	222(21.00%)	227(21.48%)	199(18.82%)
2008-2023	178(19.53%)	159(17.45%)	195(21.40%)	206(22.61%)	173(18.99%)

As shown in Table 8, it is evident that the proportion of authoritative policies has gradually declined, whereas persuasive policies have initially decreased but subsequently increased slightly. Furthermore, the proportions of persuasive, incentive, and systemic reform policies have all seen varying degrees of increase over time.

From the data presented in Table 8, a notable trend in the proportion of various policy types over time becomes visually apparent. Specifically, the proportion of authoritative policies undergoes a gradual decrease, which not only mirrors a shift away from traditional command and control methods among policymakers but also signifies a transition towards a more adaptable and diversified policy implementation approach. Concurrently, the proportion of persuasive policies undergoes a nuanced trajectory, initially declining and then subsequently rising. This initial decline may suggest a phase where policymakers were perhaps more inclined to directly intervene rather than persuade the public. However, the subsequent modest increase indicates a resurgence of persuasive policies in certain contexts, suggesting that policymakers are increasingly focusing on enhancing public understanding and acceptance through communication, education, and guidance, thereby more effectively advancing the achievement of policy goals.

Furthermore, persuasive, incentive, and systemic reform policies all exhibited upward trends over time, albeit with differing rates of growth. As previously mentioned, the expansion of persuasive policies underscores the escalating significance of communication and persuasive tactics in policy strategies. The surge in the proportion of incentive-based policies may indicate that policymakers are increasingly leveraging economic incentives, tax benefits, and other positive measures to encourage individuals or organizations to engage in policy implementation, thereby serving as an efficacious catalyst for societal transformation. Notably, the striking growth of systemic reform-oriented policies signifies not only the resolve of policymakers to tackle the underlying causes of issues and establish long-term mechanisms but also heralds a fundamental shift in policy orientation. These policies frequently entail the enhancement and innovation of institutional and legal frameworks, with the objective of optimizing resource allocation and augmenting the efficiency and efficacy of policy implementation through comprehensive and systematic adjustments, thereby laying a solid foundation for the sustainable development of society.

In summary, the alterations in the proportion of policy attitudes depicted in Table 8 not only serve as a direct mirror of policymakers’ strategic adjustments but also embody a pivotal indicator of sports advancement and policy innovation. Collectively, these shifts delineate a roadmap for the progressively flexible, diversified, and efficient evolution of physical teacher and education policy-making and implementation.

### Comparison of time distribution of policy attitude

To investigate the temporal distribution characteristics of physical teacher and education policy, we initially categorized the policy attitudes using the meticulously designed classification framework outlined in Table 6. Subsequently, we plotted an intricate broken line graph by setting the time axis as the horizontal coordinate and the cumulative count of various policy attitudes as the vertical coordinate. This resulted in the time distribution map of physical teacher and education policy attitudes, as depicted in Fig 10. This chart not only visually represents the data but also provides a profound analysis of the underlying context of policy shifts.

**Fig 11:**
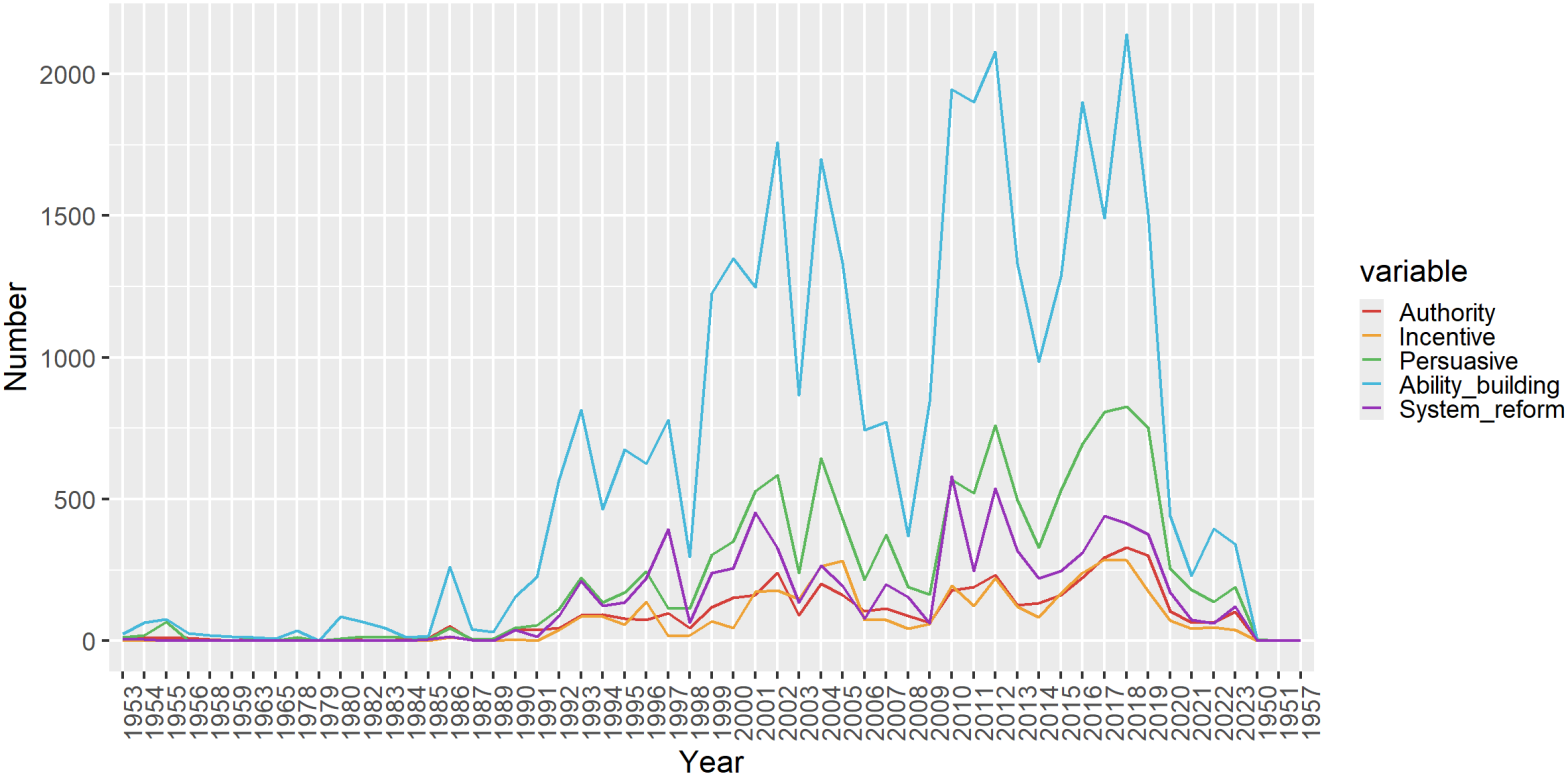
Policy attitudes in different years.

A particularly notable observation from Fig 11 is that the number of ability-building policy attitudes consistently dominates across all categories, with this advantage becoming even more evident since 1990. This trend underscores not only the significant emphasis that physical teacher and education policy has placed on the training and development of physical teachers’ abilities throughout the policymaking process, but also reflects policymakers’ aspirations to elevate the professional quality of teachers to steer sports development in a healthier and more sustainable direction. The consistent reinforcement of ability-building policies has undoubtedly provided robust support for enhancing the overall quality of physical education teachers, while also laying a solid foundation for the future progress of physical education.

Subsequently, persuasive policy attitudes, while fewer in number compared to ability-building attitudes, still hold a significant position across all categories. By employing methods of guidance and persuasion, persuasive policies aim to ignite the internal motivation of physical education teachers and foster their recognition of educational concepts and the deepening of practical implementation. The presence of this policy attitude not only diversifies the strategic approaches to policy implementation but also enhances the flexibility and relevance of policy execution.

The third category is the systematic reform policy, which is centered on optimizing and upgrading the entire physical education system. Its objective is to drive an overall enhancement of education quality through systemic reforms. Although its quantity is less than the first two types of attitudes, each phase of the reform embodies a profound understanding of the future of education and forward-thinking planning. In contrast, the number of incentive-based and authoritative policy attitudes is relatively scant. However, it is noteworthy that the number of these two types of policy attitudes exhibits a gradual upward trend over time. This signifies that, while adhering to principles and boundaries, policymakers are continually exploring more flexible and effective incentive mechanisms. Their aim is to respect teachers’ autonomy, thereby further igniting their enthusiasm and fostering innovation.

## Discussion

### Conclusion

This study employs text mining, topic modeling, and visualization techniques to systematically analyze 573 national-level physical education (PE) teacher and teaching policies in China from 1950 to 2023. The findings reveal the evolutionary logic of policy themes, attitude orientations, and issuing institution networks, offering novel insights into the historical dynamics and future directions of China’s PE policy development.

**Evolution of physical teacher and education policy themes: from single-focus to diversified refinement.** Physical teacher and education policy themes shifted from emphasizing physical teachers’ basic qualities, to professional skills, and finally to comprehensive quality and innovation—adapting to national educational reform and social needs. The early stage (1950–1977) focused on foundational physical frameworks (e.g., mandatory curricula) to rebuild post-founding education systems. The adjustment stage (1978–2007) prioritized professionalization, driven by reform and opening-up and Western educational integration, seen in policies like Guidelines for the Teaching of Sports Courses. Post-2008, the focus on comprehensive development aligned with quality-oriented education and the Beijing Olympic Games’ legacy, reflecting a move from general teacher groups to individual professional growth.

**Physical teacher and education policy attitude transformation: rigidity to flexibility** Authoritative policies declined (23.64% → 19.53%), while persuasive (21.40%), incentive (17.45%), and ability-building (22.61%) attitudes rose—shifting governance from top-down control to collaborative guidance.

**Issuing institutions: toward synergistic governance** Jointly issued policies rose (9.84% → 32.36%), with participating institutions peaking at 14—ending fragmented “multi-door” policy-making. The Ministry of Education (MOE) led (489/573 policies), while the Ministry of Finance (MOF), National Development and Reform Commission (NDRC), and Ministry of Human Resources and Social Security (MOHRSS) joined—covering funding, resources, and teacher welfare.

### Limitations and future research

Limitations include focusing only on national policies (excluding regional variations), analyzing texts not implementation outcomes, and potential LDA oversimplification of themes. Future work could incorporate regional data, combine quantitative analysis with teacher interviews/case studies, extend the timeframe post-2023, and compare China’s policies internationally.

## Supporting information

S1 File Template data collection form. (XLSX)

S2 File Data extracted from the included studies. (XLSX)

S3 File Subject headings for clustering. (XLSX)

S4 File Filter words manually. (XLSX)
